# Indications for a Central Role of Hexokinase Activity in Natural Variation of Heat Acclimation in *Arabidopsis thaliana*

**DOI:** 10.3390/plants9070819

**Published:** 2020-06-29

**Authors:** Vasil Atanasov, Lisa Fürtauer, Thomas Nägele

**Affiliations:** LMU Munich, Plant Evolutionary Cell Biology, Großhaderner Str. 2-4, 82152 Planegg, Germany; Vasil.Atanasov@campus.lmu.de (V.A.); lisa.fuertauer@lmu.de (L.F.)

**Keywords:** photosynthesis, carbohydrate metabolism, hexokinase, heat acclimation, environmental changes, natural variation, high light, combined stress

## Abstract

Diurnal and seasonal changes of abiotic environmental factors shape plant performance and distribution. Changes of growth temperature and light intensity may vary significantly on a diurnal, but also on a weekly or seasonal scale. Hence, acclimation to a changing temperature and light regime is essential for plant survival and propagation. In the present study, we analyzed photosynthetic CO_2_ assimilation and metabolic regulation of the central carbohydrate metabolism in two natural accessions of *Arabidopsis thaliana* that originate from north western Russia and south Italy during exposure to heat and a combination of heat and high light. Our findings indicate that it is hardly possible to predict photosynthetic capacities under combined stress from single stress experiments. Further, capacities of hexose phosphorylation were found to be significantly lower in the Italian than in the Russian accession, which could explain an inverted sucrose-to-hexose ratio. Together with the finding of significantly stronger accumulation of anthocyanins under heat/high light, these observations indicate a central role of hexokinase activity in the stabilization of photosynthesis and carbohydrate metabolism during environmental changes.

## 1. Introduction

Changes of growth temperature and light intensity broadly affect plant molecular, physiological, and developmental processes. Hence, plant acclimation to changing environmental conditions is essential for stabilizing growth, development, and yield production. The genetic model plant *Arabidopsis thaliana* represents an attractive system for studying acclimation capacities to environmental conditions in context of geographical origin. The availability of numerous natural accessions from a wide range of geographical origin enables the comparison of acclimation strategies and stress-responses on a genetic, biochemical, physiological and ecological level [1,2,3,4]. Such comparative approaches potentially provide detailed molecular insight into the complex regulation of plant metabolism, which promotes our understanding of how global climatic changes affect plant communities and ecosystems and might indicate breeding strategies to increase stress tolerance of crop plants. As outlined before, it is necessary to study plant acclimation and response to an abiotic stress combination because it remains hardly possible to reliably predict specific plant responses from single stress experiments [5,6].

Estimates of how global warming will affect ecosystems is a central task of current (plant) biological research. Heat affects plant metabolism and metabolic regulation by its impact on enzyme activities, membrane composition and rigidity, and signaling cascades [7,8]. Heat stress typically results in increased generation of reactive oxygen species (ROS), which can cause membrane damage, induce cytotoxic free-radical reactions, and impact on redox regulation and cellular signaling [9]. Subjecting plants to heat stress induces a series of *heat stress-responsive* (*HSR*) genes, encoding proteins like chaperones and ROS scavengers, which stabilize cellular processes, and are critical for plant thermotolerance [9].

Photosynthetic activity has been reported over a wide range of temperatures, and photosynthetic acclimation capacity to changing temperature regimes may vary substantially between species [10,11]. For example, a comparison of *Arabidopsis thaliana*, accession Columbia, to *Brassica oleracea* var. *acephala* revealed a larger capacity of *B. oleracea* for the acclimation of photosynthesis to moderate changes in growth temperature than of *A. thaliana* [10]. Under moderately elevated temperatures, i.e., between 35 and 40 °C, net photosynthetic rates typically decrease, which can be explained by deactivation of Rubisco (Ribulose-1,5-bisphosphate carboxylase/ oxygenase) while PSII (photosystem II) function is not affected [12,13]. Enzyme activities that were involved in regeneration of ribulose bisphosphate, e.g., phosphoribulokinase, glyceraldehyde 3-phosphate dehydrogenase and sedoheptulose-1,7-bisphosphatase were found to be decreased under heat which implied a direct effect on the central carbohydrate metabolism [14,15,16,17]. Metabolome analysis in *Arabidopsis* showed that a majority of heat-responsive metabolites are shared with cold shock response [18]. When comparing diverse abiotic stress responses revealed that sucrose, the central transport sugar in many plant species and an important energy and signaling molecule [19], accumulated under many stress conditions [20]. However, although sucrose accumulation represents a well-known and conserved stress response, many aspects of underlying metabolic regulation remain unclear. Among others, this is due to the central metabolic and regulatory role of sucrose metabolism in many biochemical and physiological reactions [21]. 

We compared response of two natural accessions to moderate heat (32 °C) to elucidate effects and regulation of photosynthetic CO_2_ assimilation and carbohydrate metabolism under changing environmental conditions. Accessions originated from Sicily/Italy (Ct-1) and north western Russia (Rsch-4). Both accessions were chosen due to the different profile of mean daily temperatures during the year at their geographical origin ranging between 15/33 °C (Catania, Ct-1) and −7/23 °C (Tver Oblast; Rsch-4; source: WMO World Weather Information Service (https://worldweather.wmo.int)). Based on the finding that Ct-1 had transiently higher capacity to fix CO_2_ under heat and high light intensities, we further combined moderate heat with high light exposure and monitored the dynamics of the central carbohydrate metabolism.

## 2. Results

Photosynthesis and carbohydrate metabolism are immediately affected by changes in temperature and/or light intensity. Hence, rates of net photosynthesis (NPS) were recorded together with relative changes of carbohydrate, protein, and anthocyanin concentrations and enzyme activities of the central carbohydrate metabolism to estimate effects of elevated temperature and high light intensities on natural *Arabidopsis* accessions Rsch-4 (Russia) and Ct-1 (Italy). 

### 2.1. Light Response Curves of Net Photosynthesis Indicate Differential Heat Acclimation Capacities in Ct-1 and Rsch-4 

**Figure 1:**
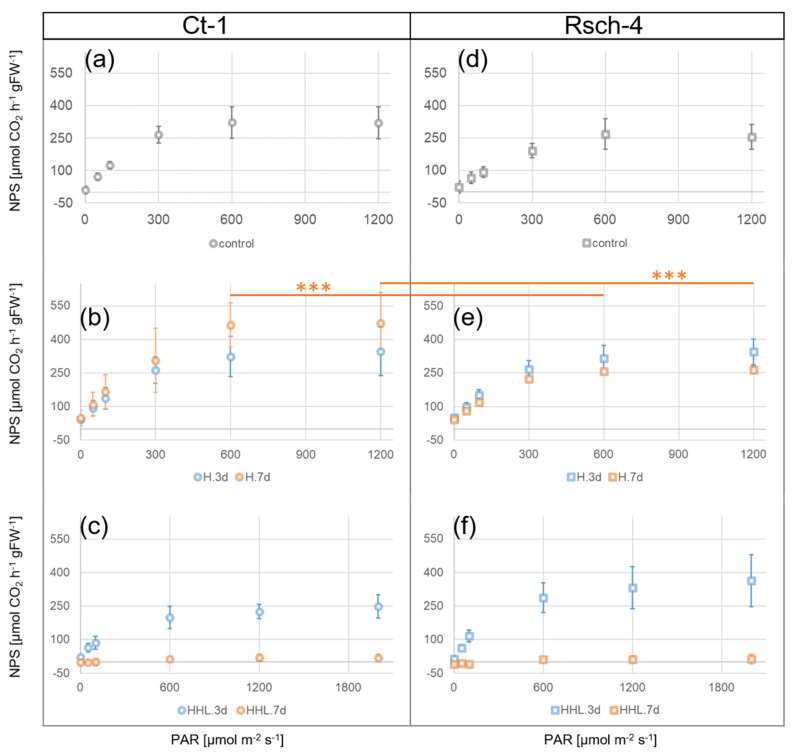
Light response curves of net photosynthesis (NPS) after exposure to heat (H) and heat/high light (HHL) for 3 days (blue) and 7 days (orange). (**a**) – (**c**) NPS of Ct-1 under (**a**) control (22 °C/100 µmol m^−2^ s^−1^), (**b**) heat (32 °C/100 µmol m^−2^ s^−1^), and (**c**) heat/high light (32 °C/600 µmol m^−2^ s^−1^). (**d**) **–** (**f**) NPS of Rsch-4 under (**d**) control (22 °C/100 µmol m^−2^ s^−1^), (**e**) heat (32 °C/100 µmol m^−2^ s^−1^), and (**f**) heat/high light (32 °C/600 µmol m^−2^ s^−1^). Asterisks indicate significance (ANOVA): *** *p* < 0.001. Circles (Ct-1) and squares (Rsch-4) represent mean values ± SD (*n* = 3–4).

Comparing the light response curves of NPS rates under control (22 °C/100 µmol photons m^−2^ s^−1^), heat (H, 32 °C/100 µmol photons m^−2^ s^−1^), and heat/high light (HHL, 32 °C/600 µmol photons m^−2^ s^−1^) revealed significant differences between Ct-1 and Rsch-4 after 7 d at 32 °C (Figure 1). The rates of NPS were significantly higher in Ct-1 than in Rsch-4 under high PAR intensity, i.e., 600 and 1200 µmol photons m^−2^ s^−1^ (Figure 1b,e). This effect was only observed after 7 d at 32 °C, not yet after 3 d. No significant effects were observed under all other tested conditions, i.e., control and HHL. However, although not significant, NPS rates in Rsch-4 were ~1.5-fold higher after 3 d HHL and this effect was most pronounced under high photosynthetically active radiation (PAR), i.e., >600 µmol photons m^−2^ s^−1^ (Figure 1c,f). After 7 d of HHL, leaves became senescent (not shown) and NPS rates of both accessions dropped below 25 µmol CO_2_ h^−1^ gFW^−1^ across all PAR intensities (Figure 1c,f).

### 2.2. Sucrose-Hexose Dynamics Indicate Differential Metabolic Regulation in Ct-1 and Rsch-4 during HHL Exposure

**Figure 2:**
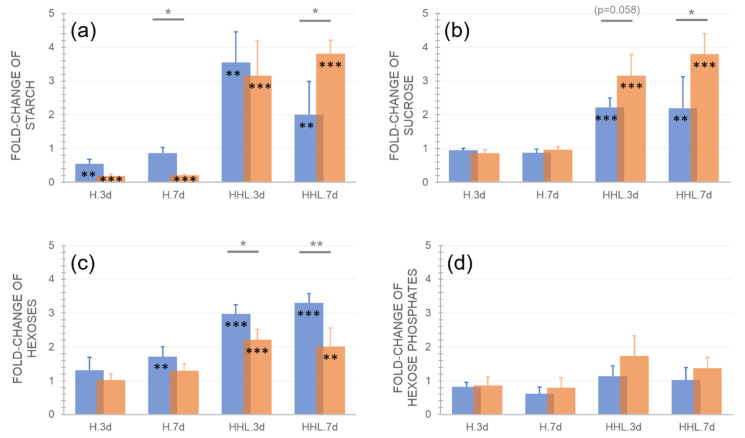
Relative changes of central carbohydrates after exposure to heat (H) and heat/high light (HHL). Fold-change of (**a**) starch amount, (**b**) sucrose concentration, (**c**) hexoses concentration (glucose + fructose), and (**d**) hexose phosphate concentration (glucose 6-P + fructose 6-P) as compared to control (i.e., a fold-change of 1 indicates no change compared to control). Blue bars: Ct-1, orange bars: Rsch-4. Asterisks indicate significance (ANOVA), grey asterisks for comparison between accessions, black asterisks for comparison of treatment to control: * *p* < 0.05; ** *p* < 0.01; *** *p* < 0.001. Bars represent mean values ± SD (*n* = 5).

Exposure to H and HHL had a significantly different impact on the central carbohydrate metabolism of Ct-1 and Rsch-4. The starch amount was significantly decreased in both accessions under heat and this effect was stronger in Rsch-4 than in Ct-1 (Figure 2a). After 7 d at 32 °C, starch amount in Ct-1 did not differ significantly when compared to control condition while the starch amount in Rsch-4 remained significantly lower than under 22 °C. Under HHL, both accessions accumulated significantly more starch than under control, and after 7 d starch amount in Rsch-4 was significantly higher than in Ct-1. Sucrose concentration remained unaffected in both accessions under H while it accumulated two-fold in Ct-1 and three-fold in Rsch-4 under HHL (Figure 2b). Rsch-4 accumulated significantly more sucrose than Ct-1 if heat was combined with high light. Reciprocally, hexoses, i.e., glucose and fructose, accumulated to a significantly higher concentration in Ct-1 than in Rsch-4 under HHL (Figure 2c). For hexoses phosphates, no significant accumulation was observed in either tested condition (Figure 2d).

### 2.3. Accessions Differ in Their Protein and Anthocyanin Concentration under HHL 

**Figure 3:**
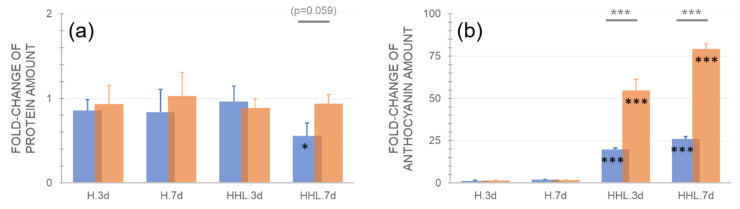
Relative changes of protein amount and anthocyanin concentration after exposure to heat (H) and heat/high light (HHL). Fold-change of (**a**) protein amount, (**b**) anthocyanin concentration compared to control (i.e., a fold-change of 1 indicates no change compared to control). Blue bars: Ct-1, orange bars: Rsch-4. Asterisks indicate significance (ANOVA), grey asterisks for comparison between accessions, black asterisks for comparison of treatment to control: * *p* < 0.05; ** *p* < 0.01; *** *p* < 0.001. Bars represent mean values ± SD (*n* = 5).

The protein amount did not differ between the control and treatment samples, except for Ct-1 after 7 d HHL where the protein amount was significantly decreased (Figure 3a). While anthocyanin concentration neither significantly changed under heat, combination with high light (HHL) resulted in a strong (>20-fold) and significant increase in both accessions (Figure 3b; *p* < 0.001, ANOVA). Already after 3 d HHL, anthocyanin concentration increased more than two-fold higher in Rsch-4 compared to Ct-1 and more than 3-fold after 7 d HHL (Figure 3b). 

### 2.4. Effects of H and HHL on Enzyme Activities of the Central Carbohydrate Metabolism

**Figure 4:**
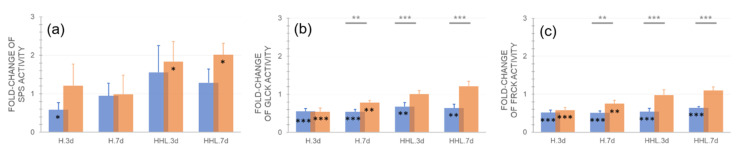
Relative changes of enzyme activities after exposure to heat (H) and heat/high light (HHL). Fold-change of (**a**) sucrose phosphate synthase (SPS) activity, (**b**) glucokinase (Glck) activity, and (**c**) fructokinase (Frck) activity compared to control (i.e., a fold-change of 1 indicates no change compared to control). Blue bars: Ct-1, orange bars: Rsch-4. Asterisks indicate significance (ANOVA), grey asterisks for comparison between accessions, black asterisks for comparison of treatment to control: * *p* < 0.05; ** *p* < 0.01; *** *p* < 0.001. Bars represent mean values ± SD (*n* = 5).

In Rsch-4, HHL resulted in a significant increase of sucrose phosphate synthase (SPS) activity (Figure 4a). Additionally, in Ct-1 SPS activity increased under HHL compared to control conditions but less significantly. After 7 d HHL, Rsch-4 had significantly doubled its SPS activity while no significant change was observed for Ct-1. Exposure to 3 d H resulted in a significant decrease of SPS activity in Ct-1 (~50% of control) and this effect disappeared after 7 d H. The SPS activity of Rsch-4 was not significantly affected by heat exposure. Gluco- and fructokinase (GlcK/FrcK) activity was significantly reduced in Ct-1 by 40–50% under H and HHL (Figure 4b,c). In Rsch-4, both GlcK and FrcK activity was significantly reduced under H, yet not under HHL, when compared to control.

**Figure 5:**
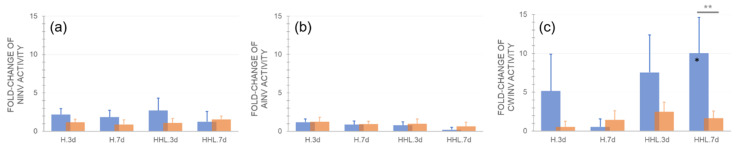
Relative changes of invertase activities after exposure to heat (H) and heat/high light (HHL). Fold-change of (**a**) neutral invertase (nInv) activity, (**b**) acidic invertase (aInv) activity, and (**c**) cell wall invertase (cwInv) activity compared to control (i.e., a fold-change of 1 indicates no change compared to control). Blue bars: Ct-1, orange bars: Rsch-4. Asterisks indicate significance (ANOVA), grey asterisks for comparison between accessions, black asterisks for comparison of treatment to control: * *p* < 0.05; ** *p* < 0.01. Bars represent mean values ± SD (*n* = 5).

The activity of neutral invertase (nInv) doubled in Ct-1 when exposed to H and HHL but this effect was not significant (Figure 5a). Acidic invertase (aInv) was least affected by H and HHL in both accessions (Figure 5b), while cell wall invertase (cwInv) was increased up to 10-fold in Ct-1 under HHL (Figure 5c). Due to relatively high variance of cwInv activities in Ct-1 most differences between control and treatment and between both accessions were not significant, although the mean values indicated a strong treatment effect in Ct-1. In general, nInv and cwInv activity in Rsch-4 was less affected by H and HHL than in Ct-1.

## 3. Discussion

The regulation of plant metabolism under a changing temperature regime comprises and affects numerous pathways, enzymatic reactions, and signaling cascades. Previous studies have shown that many compounds of primary metabolism show similar dynamics under heat and cold stress. For example, coordinated increases of amino acids derived from pyruvic acid or oxaloacetate, precursors of polyamines, and compatible solutes were observed under both high and low temperatures [18]. The stabilization of a metabolic homeostasis under changing temperature regimes essentially depends on tight regulation of enzyme activities in biosynthesis and degradation pathways. Although temperature was only moderately increased in the present study from 22 °C to 32 °C, velocities of enzyme-catalyzed reactions typically increase two to three-fold per each 10 °C [22,23]. Consequently, transcriptional, translational, post-translational, and metabolic regulation needs to be reprogrammed to stabilize metabolism and prevent the over-accumulation of ROS under elevated temperature [9]. In the present study, heat acclimation of two natural accessions, Ct-1 and Rsch-4, was analyzed after 3 d and 7 d exposure to 32 °C. Light response curves of NPS rates under 32 °C were found to be similar, or even slightly elevated, as compared to control measurements under 22 °C. This indicates that, independent of geographical origin, *Arabidopsis* plants are able to photosynthetically acclimate to moderate heat at least within three days. These findings are similar to a previous study, which showed a slight increase of NPS rates in *Arabidopsis thaliana*, accessions Columbia, between 20 °C and 30 °C [10]. Yet, under high PAR within light response curves, Ct-1 was observed to have significantly higher NPS rates than Rsch-4 after seven days of heat acclimation (see Figure 1b,e). The accession Ct-1 originates from Sicily/Italy where transient high PAR intensities under high temperature may occur more often than in western Russia, which represents the geographical origin of Rsch-4. However, after exposure to high temperature and high light for 3 d and 7 d, no significant difference was observed anymore between the NPS rates of both accessions (see Figure 1) indicating that acclimation capacity to such environmental conditions cannot be reliably predicted from transient measurements. Such limited predictability of combined stress and acclimation response has been reported before and it has also been shown for combination of abiotic and biotic stress [24,25]. After 7 d of exposure to HHL NPS rates dropped dramatically in both accessions. Hence, although Rsch-4 and Ct-1 both could stabilize NPS rates until 3 d of HHL to a similar extent, like under control and heat, 7 d of combined stress resulted in severe reduction of photosynthetic CO_2_ fixation which was accompanied by strong accumulation of anthocyanins in both accessions. Interestingly, although we could not detect any significant difference in NPS rates (normalized to leaf fresh weight) after 7 d HHL between both accessions, only Ct-1 was significantly affected in protein amount compared to control which might indicate a more advanced level of senescence than in Rsch-4 which is typically accompanied by protein degradation [26]. Thus, comparing differential acclimation response of Rsch-4 and Ct-1 might also be of interest for further molecular studies focusing, e.g., on regulation of phytohormones, anthocyanin metabolism, and photoreceptors, due to differential capabilities of inducing secondary metabolism and stabilization of protein amount [27].

Although light response curves revealed similar NPS rates in both accessions, the metabolism of starch and soluble carbohydrates, i.e., sucrose and hexoses, was found to significantly differ between Ct-1 and Rsch-4. While the sucrose concentration did not vary significantly under heat in both accessions, starch was significantly decreased after 3 d and 7 d in Rsch-4, and it reached the control amount again after 7 d in Ct-1. Starch amount has previously been reported to decrease in accession Columbia-0 during heat exposure, which is also confirmed by our data [28]. However, differential starch dynamics between both accessions after 7 d at 32 °C indicate that heat acclimation in Ct-1 results in recovery of starch metabolism while it remains affected in Rsch-4. From the presented data, it also remains to be elucidated whether diurnal starch dynamics in Ct-1 are similar to control or if diurnal rates of starch synthesis and degradation are dampened or enhanced after 7 d at 32 °C.

Neither sucrose nor hexose concentration were significantly affected during heat exposure, except for Ct-1, which accumulated ~1.5-fold hexose concentration after 7 d at 32 °C. This finding contrasts previous reports, which have shown a significant doubling of sucrose under heat [24]. This discrepancy might be explained by differential growth conditions, which were long day (16/8 h) in the present study and 12/12 h in the study of Prasch and colleagues [24]. Further, Prasch et al. used *Arabidopsis* accession Columbia for their analyses, which might induce further accession-specific variance in heat acclimation mechanisms.

**Figure 6:**
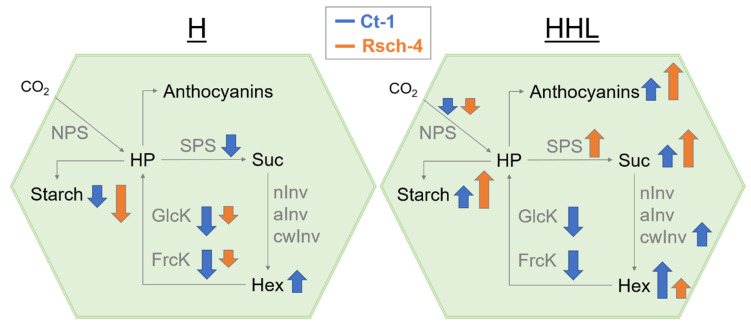
Molecular responses of Ct-1 (blue) and Rsch-4 (orange) under moderate heat (H, left side) and heat/high light (HHL, right side). Only significant effects are shown and size of arrows (up or down) are proportional to fold-changes compared to control samples (increase or decrease). HP: hexose phosphates; Suc: sucrose; Hex: hexoses; NPS: net photosynthesis; SPS: sucrose phosphate synthase; nInv: neutral invertase; aInv: acidic invertase; cwInv: cell wall invertase; GlcK: glucokinase; FrcK: fructokinase.

Most significant differences between Ct-1 and Rsch-4 were detected for gluco- and fructokinase activities during of H and HHL exposure. Particularly under HHL, both hexose phosphorylating activities remained significantly lower in Ct-1 than in Rsch-4. Simultaneously, hexoses accumulated to significantly higher concentration in Ct-1 than in Rsch-4, which rather accumulated sucrose under HHL (Figure 6).

These findings indicate a central role of hexose phosphorylation reactions in accession-specific regulatory strategies of carbohydrate metabolism under moderate heat. Although fold-change of SPS activity did not significantly differ between both accessions, Rsch-4 increased its activity under 32 °C slightly more than Ct-1, which supports the hypothesis of two different heat acclimation strategies, which are either sucrose accumulating (Rsch-4) or hexose accumulating (Ct-1). Further, aInv and cwInv activity were higher in Ct-1 than in Rsch-4, and this difference became the most significant for cwInv activity after 7 d HHL. In summary, these observations suggest that in Ct-1 more pronounced accumulation of hexoses occurred due to (i) lower hexose phosphorylation rates, and (ii) higher sucrose cleavage rates. Enzymatic reactions catalyzed by SPS, GlcK and FrcK, and invertases constitute a futile cycle of sucrose biosynthesis and degradation which has previously been discussed in context of metabolic stabilization under environmental changes, e.g., low temperature [29,30,31]. In context of recent findings, which provided evidence for central metabolic role of hexokinase 1 in response to high light stress [32], it remains tempting to speculate that differential GlcK and FrcK activities in Ct-1 and Rsch-4 explain different subcellular sucrose cycling capacities resulting in different stress acclimation capacities. However, in the present study no significant difference was detected between NPS rates of Ct-1 and Rsch-4, which suggests similar capacities of both accessions to photosynthetically acclimate to H and HHL. It remains to be tested in future studies whether different acclimation capacities can be detected between 3 d and 7 d of HHL before NPS rates drop dramatically, or if other measures for heat acclimation capacity, e.g., leakage or survival assays, may correlate with sucrose cycling capacity. Finally, resolving plant metabolism to a subcellular level and the estimation of enzymatic reaction rates by kinetic modelling will reveal whether sucrose cycling represents a conserved mechanism of stabilization of plant metabolism in a changing environment.

## 4. Materials and Methods 

### 4.1. Plant Material and Growth Conditions

Seeds of natural *Arabidopsis thaliana* accessions Ct-1 (Sicily/Italy; latitude 37.3°, longitude 15°; 1001 Genomes Id: 7067) and Rsch-4 (Russia; latitude 56.3°, longitude 34°; 1001 Genomes Id: 7322) were incubated in tap water in darkness at 4 °C for two days before sowing. Plants for molecular analysis were grown in a randomized design in 9 × 9 cm pots on a 1:1 mixture of GS90 soil and vermiculite with four plants per pot. For photosynthesis measurements, plants were grown in round pots with diameter 7 cm. All of the plants were grown for 28 days under short day conditions (8 h/16 h light/dark, 22 °C/18 °C, 100 µmol photons m^−2^ s^−1^, 60% humidity) before transfer to long day growth conditions for further 14 days (16 h/8 h light/dark). Plant material was sampled at midday, i.e., after 8 h in the light. For heat treatment (H), plants were exposed to 32 °C/100 µmol photons m^−2^ s^−1^ for 16 h and 28 °C during the night (8 h). Heat/high light samples (HHL) were exposed to 32 °C/600 µmol photons m^−2^ s^−1^ for 16 h and 28 °C during the night (8 h). Plants of all conditions were grown in the same growth cabinet (Conviron^®^, Adaptis A1000-AR, www.conviron.com). One sample consisted of four complete leaf rosettes, which were immediately snap frozen in liquid nitrogen and stored at −80 °C until further use. For each accession (Ct-1 and Rsch-4), treatment (control, H and HHL) and time point (3 d and 7 d), five replicates were analyzed (*n* = 5), each consisting of four leaf rosettes from four different pots that were randomized within the growth cabinet.

### 4.2. Quantification of CO_2_ Assimilation Rates (Net Photosynthesis, NPS)

The plants were dark incubated for 15 min before intensity of photosynthetically active radiation (PAR) was stepwise increased in intervals of five min from 0 to 50, 100, 300, 600, and 1200 µmol photons m^−2^ s^−1^. For plants that were grown under HHL, PAR intensities were 0, 50, 100, 600, 1200, and 2000 µmol photons m^−2^ s^−1^. All of the measurements were performed within the growth cabinet (Conviron^®^, Adaptis A1000-AR, Winnipeg, Canada. www.conviron.com) under 22 °C (control) or 32 °C (H and HHL). Ambient air CO_2_ concentration was 410 ppm. All of the measurements were performed using a WALZ^®^ GFS-3000FL system that was equipped with measurement head 3010-S, including an LED-array to set PAR intensities within light response curves (Heinz Walz GmbH, Effeltrich, Germany, https://www.walz.com/). For a measurement, one leaf of a rosette was (non-invasively) clamped into the measurement head. The replicates represent independent measurements on different plants.

### 4.3. Quantification of Carbohydrate, Anthocyanin and Protein Amounts

Relative starch amount was determined as described earlier with slight modification [33]. Frozen and ground plant material were incubated with 400 µl of 80% EtOH at 80 °C for 30 min. The samples were centrifuged, supernatants were transferred to a new tube, and the extraction was repeated. Supernatants were unified and dried in a desiccator and the dried pellet was used for relative quantification of sugars. Starch was hydrolysed using 500 µl 0.5 M NaOH at 95 °C for 60 min. The samples were then slightly acidified with 500 µl 1 M CH_3_COOH before digestion with amyloglucosidase for 2 h at 55 °C. The amount of released glucose moieties from starch digestion was photometrically determined by a glucose oxidase/peroxidase/o-dianisidine reaction at 540 nm.

Relative concentrations of glucose, fructose, and sucrose were determined from dried EtOH extracts. Sucrose was quantified applying an anthrone assay after incubation with 30% KOH at 95 °C. Anthrone was dissolved in 14.6 M H_2_SO_4_ (0.14% w/v), incubated with the samples for 30 min. at 40 °C, and absorbance was determined at 620 nm. 

Glucose concentration was determined using a coupled hexokinase/glucose 6-phosphate dehydrogenase assay resulting in NADPH that was photometrically determined at 340 nm. For fructose quantification, phosphoglucoisomerase was added to the reaction mixture after glucose determination. 

Glucose 6-phosphate (G6P) and fructose 6-phosphate (F6P) were extracted and photometrically quantified using an enzyme cycling-based assay [34]. Hexose phosphates were extracted using trichloroacetic acid (TCA) in diethyl ether (16% w/v). The extracts were washed with 16% (w/v) TCA in 5 mM EGTA and neutralised with 5 M KOH/1 M triethanolamine. Enzymatic reactions catalyzed the equimolar interconversion of G6P and F6P into NADPH finally yielding a formazan dye from thiazolyl blue (MTT) which was detected photometrically at 570 nm.

The anthocyanins were extracted using 1 M HCl, first at 25 °C and a second time at 80 °C. Supernatants were pooled and the relative anthocyanin concentration was determined photometrically at 520 nm.

Proteins were extracted from plant material in 8 M urea and 50 mM HEPES-KOH (pH = 7.8). Overnight, the proteins were precipitated in acetone with 2 mM DTT, subsequently washed with methanol and acetone and again solubilized in 8 M urea, 50 mM HEPES-KOH (pH = 7.8). Protein amount was determined photometrically using the Bradford assay [35]. 

### 4.4. Quantification of Enzyme Activities

Sucrose phosphate synthase (SPS) activity was determined under substrate saturation using the anthrone assay as described earlier [33]. Frozen leaf tissue was ground to a fine powder using mortar and pestle. Ground material was then suspended in extraction buffer containing 50 mM HEPES-KOH (pH 7.5), 10 mM MgCl_2_, 1mM EDTA, 2.5 mM DTT, 10% (v/v) glycerine, and 0.1% (v/v) Triton-X-100. After incubation on ice, the extracts were further incubated for 30 min. at 25 °C with reaction buffer containing 50 mM HEPES-KOH (pH 7.5), 15 mM MgCl_2_, 2.5 mM DTT, 35 mM UDP-glucose, 35 mM F6P, and 140 mM G6P. Reactions were stopped by adding 30% KOH and heating the samples to 95 °C. Sucrose was photometrically determined after incubation with anthrone in H_2_SO_4_, as described above.

Gluco- and fructokinase activities were determined photometrically at 340 nm recording the slopes of NADP^+^ reduction to NADPH + H^+^ [36]. Ground and frozen leaf tissue was suspended in extraction buffer containing 50 mM Tris-HCl (pH 8.0), 0.5 mM MgCl_2_, 1 mM EDTA, 1 mM DTT and 1% (v/v) Triton-X-100. The samples were incubated on ice for 15 min. before. After centrifugation at 4 °C, the reaction was started using the supernatant and adding reaction buffer containing 100 mM HEPES-KOH (pH 7.5), 10 mM MgCl_2_, 2 mM ATP, 1 mM NADP, 0.5 U G6PDH, and either 5 mM glucose for glucokinase measurement or 5 mM fructose and 0.5 U PGI for fructokinase measurement.

Activities of neutral (nInv), acidic (aInv) and cell wall-bound (cwInv) invertase under substrate saturation were determined as described before with slight modifications [37]. Frozen and ground leaf tissue incubated with extraction buffer containing 50 mM HEPES-KOH (pH 7.5), 5 mM MgCl_2_, 2 mM EDTA, 1 mM phenylmethylsulfonylfluoride (PMSF), 1 mM DTT, 10% (v/v) glycerine, and 0.1% (v/v) Triton-X-100. After incubation on ice, the samples were centrifuged at 4 °C before supernatants were transferred to a new tube. Pellets contained cell wall-bound invertase and they were re-suspended in extraction buffer. The activity of soluble invertases, i.e., nInv and aInv, were determined from supernatants. For activity measurement of nInv a reaction buffer was composed of 20 mM HEPES-KOH (pH 7.5) and 100 mM sucrose), while for aInv and cwInv activity measurements the reaction buffer contained 20 mM sodium acetate (pH 4.7) and 100 mM sucrose. After incubation of extract with reaction buffer, the reaction was stopped at 95 °C and glucose built from invertase reactions was photometrically determined at 540 nm while using a coupled glucose oxidase/peroxidase/o-dianisidine assay.

### 4.5. Statistics

For statistics, each replicate of a measurement under stress (H or HHL) was divided by the corresponding mean value that was measured under control condition to determine fold-changes. All (relative) metabolite concentrations, protein amounts, and enzyme activities were normalized to fresh weight before calculation of fold-changes. Statistical data analysis was performed using the free software environment R Version 3.6.1 (www.r-project.org/) [38] and RStudio Version 1.2.5019 (https://rstudio.com/) [39]. Statistics comprised an analysis of variance (ANOVA) and Tukey’s honestly significant difference post-hoc test. Data were visualized using Microsoft Excel (Microsoft Corporation, Redmond, WA, USA; www.microsoft.com).
